# Evaluation of the Effects of Dental Implant Dimension and Design on Primary Implant Stability Using Different Stability Measurement Devices

**DOI:** 10.3390/jcm15218626

**Published:** 2026-11-05

**Authors:** Tuna Sumer, Nihat Akbulut

**Affiliations:** 1 Department of Oral and Maxillofacial Surgery, Faculty of Dentistry, Institute of Graduate Studies, Ondokuz Mayis University, Kurupelit, Samsun 55139, Turkey; 2 Department of Oral and Maxillofacial Surgery, Faculty of Dentistry, Ankara University, Mevlana Blv. Yenimahalle, Ankara 06560, Turkey; drnihatakbulut@yahoo.com

**Keywords:** bone density, dental implants, primary stability, resonance frequency analysis

## Abstract

**Background/Objectives:** Dental implants are an established treatment for functional and aesthetic rehabilitation of partially or completely edentulous patients. This in vitro study evaluates the effects of dental implant design, diameter, length, and bone density on primary implant stability using Osstell ISQ and Penguin Resonance Frequency Analysis (RFA) devices. **Materials and Methods:** A total of 96 dental implants were included in this study. Combinations of two implant designs (T6 Standard and T6 Torq), two implant diameters (3.5 mm and 5.0/4.8 mm), and two implant lengths (8 mm and 14 mm) were placed into synthetic bone blocks with densities of PCF15 and PCF30. Implant stability was measured twice from two different directions using Osstell ISQ and Penguin RFA devices. The data were statistically analyzed. **Results:** Both RFA devices demonstrated high measurement reproducibility. When all measurements were evaluated collectively, the intraclass correlation coefficient (ICC) reached 0.999 for both devices. Bone density was identified as the most influential factor affecting primary implant stability. Significantly higher ISQ values were obtained in the high-density bone model (*p* < 0.001). Increases in implant length and diameter significantly enhanced primary stability (*p* < 0.001). T6 Torq implants exhibited significantly higher ISQ values than T6 Standard implants in low-density bone and small-dimension implant combinations (*p* < 0.001). **Conclusions:** Under the present experimental conditions, bone density showed the greatest influence on primary implant stability, followed by implant length and implant diameter. In low-density bone, particularly with short and narrow implants, more aggressive implant macrodesigns may contribute to higher primary stability. However, the clinical relevance of these findings requires further investigation.

## 1. Introduction

Dental implants have become one of the most widely accepted treatment modalities for the rehabilitation of partially and completely edentulous patients. The long-term success of implant therapy depends primarily on successful osseointegration, defined as a direct structural and functional connection between bone and implant [1,2]. Achieving adequate primary implant stability is considered a prerequisite for successful osseointegration and the predictable application of early loading protocols [3,4].

Primary stability represents the mechanical engagement between the implant and the surrounding bone immediately after placement, whereas secondary stability develops through new bone formation and remodeling during the healing process. Insufficient primary stability may compromise osseointegration and increase the risk of implant failure [2,3,5]. Primary implant stability is influenced by multiple factors, including bone quality and volume, implant macrogeometry, implant dimensions, surface characteristics, and surgical technique [1,3]. Among these variables, bone density is considered one of the most important factors affecting of primary stability. Bone quality varies according to anatomical location, with the highest bone density generally observed in the anterior mandible and the lowest in the posterior maxilla [2,6]. Adell et al. [6] reported approximately 10% higher implant success rates in the anterior mandible than in the anterior maxilla under standardized surgical and prosthetic protocols. Obtaining adequate primary stability remains challenging, particularly in low-density bone. Therefore, various implant macrodesigns continue to be developed to enhance implant stability [7,8,9]. Implants with aggressive macrodesigns, characterized by deeper and more pronounced thread geometries, have been developed to enhance mechanical engagement with the surrounding bone. Previous studies have suggested that these macrodesign modifications may improve primary stability, particularly in low-density bone [10,11]. However, the influence of implant macrodesign on primary implant stability remains controversial. Likewise, although implant diameter and implant length have been extensively investigated, their relative contributions to primary stability have not yet been fully established.

Assessment of implant stability throughout the healing period enables monitoring of the osseointegration process and may help predict the long-term prognosis of dental implants [12]. Several methods have been proposed for assessing implant stability, including histomorphometric analysis, push-out and pull-out tests, insertion torque (IT), removal torque, cutting torque, percussion tests, Periotest, and resonance frequency analysis (RFA). Among these, insertion torque provides an indirect assessment of primary stability by measuring the rotational resistance encountered during implant placement, while cutting torque reflects the resistance of bone during osteotomy preparation [13,14]. The Periotest device evaluates the damping characteristics of the implant-bone interface and expresses the results as Periotest Value (PTV) [15]. Although these methods provide valuable information regarding implant stability, each has certain limitations. RFA has become the most widely used non-invasive technique because it provides objective, reproducible, and clinically applicable measurements. RFA measures implant stability by assigning an Implant Stability Quotient (ISQ) value [8,16,17].

The aim of this in vitro study was to evaluate the effects of implant design, implant diameter, implant length, and bone density on primary implant stability and to assess the measurement repeatability of the Osstell ISQ and Penguin RFA systems. The results may contribute to a better understanding of the factors influencing primary implant stability and provide clinically relevant information for implant selection and treatment planning under different bone conditions.

## 2. Materials and Methods

A total of 96 bone-level dental implants (NucleOSS Implant System, İzmir, Türkiye) were included in the present study. The sample size was determined using an a priori power analysis performed with G*Power software (version 3.1.9.6; Franz Faul, Universitat Kiel, Germany) with the F tests—ANOVA: fixed effects, special, main effects and interactions option. Based on the effect size conventions proposed by Cohen [18], a large effect size (f = 0.40) was assumed, with a significance level of α = 0.05 and power (1-β) = 0.90. The minimum required sample size was calculated as 69. The sample size was increased by approximately 40%, resulting in a total of 96 samples (*n* = 6 per group).

Two implant macrodesigns were evaluated: T6 Standard and T6 Torq, each available in two lengths (8 and 14 mm). The T6 Standard implants were manufactured in diameters of 3.5 and 4.8 mm, whereas the T6 Torq implants were available in diameters of 3.5 and 5.0 mm. Both T6 Standard and T6 Torq implants feature a tapered body design, an internal hexagonal connection, double-threaded reverse buttress geometry, a Maxicell surface, a rounded apex, and an angled contact region. The T6 Standard design additionally incorporates cervical microthreads, whereas the T6 Torq design is characterized by deeper, cutting V-shaped threads, resulting in a more aggressive macrogeometry compared with the T6 Standard implant.

Implants were placed into rigid polyurethane foam blocks (Sawbones, Vashon, WA, USA), which simulate the mechanical properties of human trabecular bone. Two bone densities were evaluated: PCF15 (0.24 g/cm^3^), representing low-density maxillary bone, and PCF30 (0.48 g/cm^3^), representing high-density mandibular bone. All polyurethane blocks measured 13 × 18 × 4 cm.

Implant osteotomies were prepared according to the manufacturer’s recommended drilling protocol for each implant design. The final osteotomy diameters corresponded to the nominal implant diameters (3.5 and 4.8 mm for T6 Standard; 3.5 and 5.0 mm for T6 Torq), and no underpreparation was performed. In the high-density polyurethane blocks (PCF30), an additional hard-bone drill was used according to the manufacturer’s recommendations.

Two polyurethane blocks were used, one for each bone density, with 48 implants placed in each block. To standardize implant positioning, angulation, and inter-implant spacing, a custom-designed three-dimensionally printed surgical guide was used. The guide was designed with implant sites positioned at the centers of 20 × 20 mm squares, ensuring a minimum distance of 15 mm between adjacent implants.

**Figure 1:**
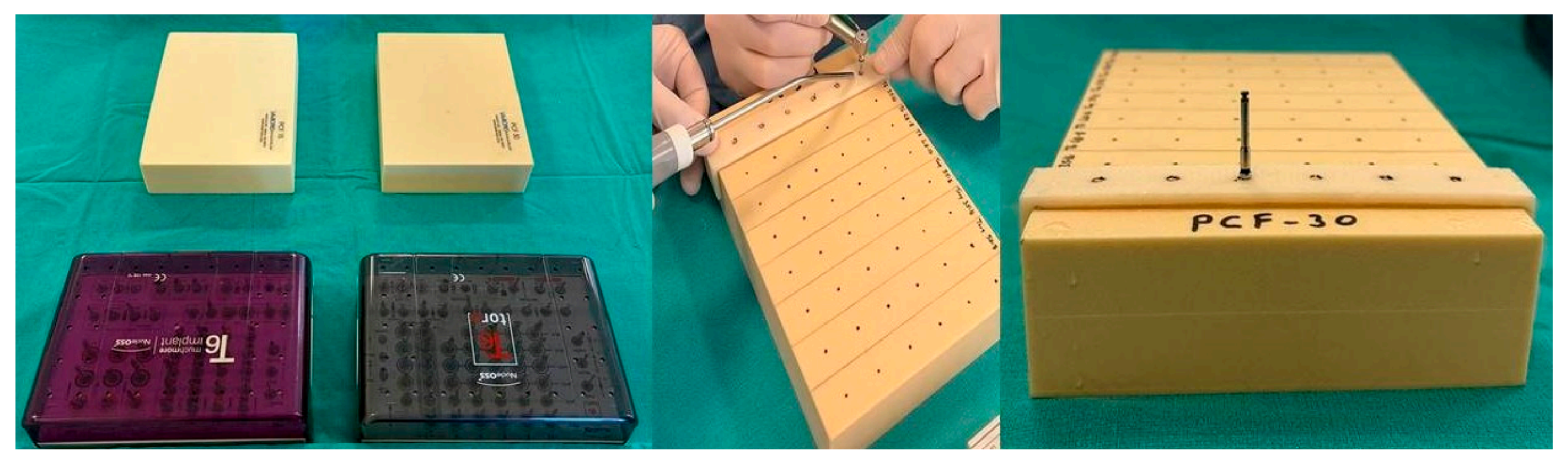
Bone blocks and surgical kits used in this study and preparation of implant osteotomy sites.

Following osteotomy preparation, all implants were placed by the same investigator under standardized experimental conditions. Each combination of implant design, diameter, and length was represented by six implants in each bone-density group (Figure 1).

Primary implant stability was evaluated after implant placement using two commercially available RFA devices: Osstell ISQ (Osstell AB, Gothenburg, Sweden) and Penguin RFA (Integration Diagnostics Sweden AB, Gothenburg, Sweden). New, original, reusable screw-type transducers (SmartPeg Type 21 and MulTipeg 66) specifically manufactured for the Nucleus implant system were used for all measurements. The transducers were manually tightened to the implants by the same investigator.

**Figure 2:**
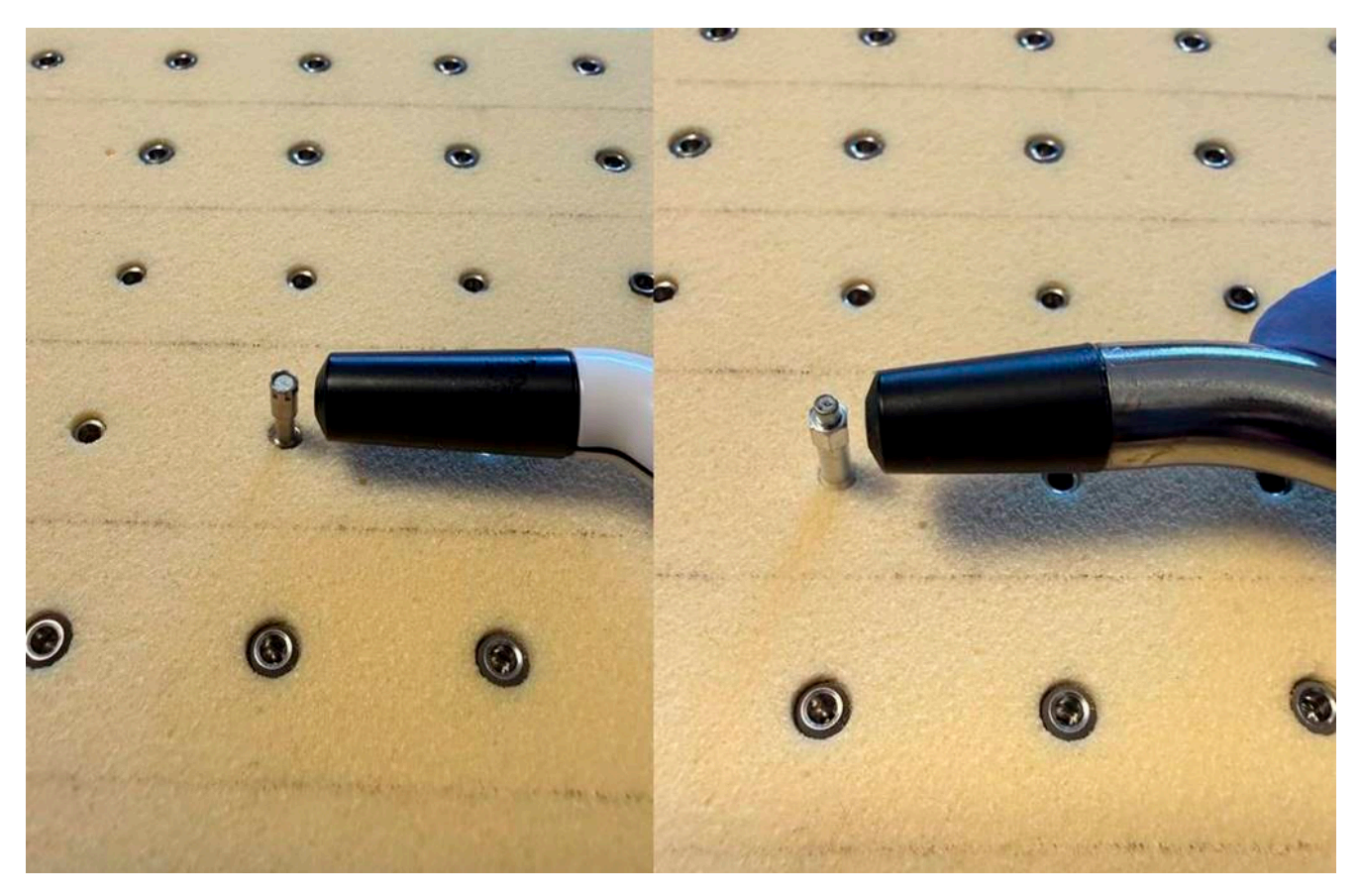
ISQ measurements with Osstell ISQ and Penguin RFA.

ISQ measurements were performed in a standardized sequence, with Osstell ISQ used first, followed by Penguin RFA. For each device, two measurements were obtained in both the buccal and lingual directions. During measurement, the probe was positioned approximately 2 mm from the transducer at an angle of approximately 90°. All measurements were recorded as ISQ values and were performed by the same investigator under identical environmental conditions (Figure 2).

Statistical analyses were performed using IBM SPSS Statistics (version 27). The repeatability of repeated measurements obtained with the Osstell ISQ and Penguin RFA devices was evaluated using the intraclass correlation coefficient (ICC) based on a two-way mixed-effects model. Given the excellent repeatability observed, the first buccal measurement from each device was used for statistical analysis to ensure a standardized and consistent evaluation across all experimental groups. The normality of data distribution was assessed using the Shapiro–Wilk test. Comparisons between two independent groups were performed using the independent-samples *t*-test. Differences between Osstell ISQ and Penguin RFA measurements obtained from the same implants were evaluated using the paired-samples *t*-test. The main effects of bone density, implant design, implant diameter, and implant length, as well as their interactions on ISQ values, were analyzed separately for the Osstell ISQ and Penguin RFA datasets using factorial analysis of variance (ANOVA). Significant interactions were further explored using Duncan’s multiple comparison test. Statistical significance was set at *p* < 0.05.

## 3. Results

A total of 96 implants were evaluated, providing 768 ISQ measurements obtained using both RFA devices, with measurements performed twice in two different directions. The highest ISQ values were observed in wide-diameter, 14 mm implants placed in the high-density polyurethane blocks, whereas the lowest values were recorded in 3.5-mm-diameter, 8 mm implants placed in the low-density blocks. The overall mean ISQ value across all experimental groups was 68.2 ± 7.12. According to the RFA device used, the mean ISQ value was 68.9 ± 7.08 for the Penguin RFA system and 67.5 ± 7.13 for the Osstell ISQ system.

ICC analysis demonstrated excellent measurement repeatability for both RFA devices, with ICC values exceeding 0.99 for all repeated measurements regardless of measurement direction (Table 1). The descriptive statistics for the Penguin RFA and Osstell ISQ devices are presented in Supplementary Tables S1 and S2, respectively.

Differences in measurements between the Penguin RFA and Osstell ISQ devices were evaluated separately for 16 implant combinations using paired-samples *t*-tests. Overall, statistically significant differences between the devices were observed in 12 of the 16 implant combinations (75%), with the Penguin RFA device consistently yielding higher values in all significant comparisons.

Bone density had the greatest influence on primary implant stability. Across all experimental conditions, implants placed in PCF30 blocks consistently showed higher ISQ values than those placed in PCF15 blocks, regardless of the RFA device, implant design, diameter, or length (*p* < 0.001). Examination of the effect sizes revealed consistently large effects across all combinations (Table 2).

When the effect of implant length on ISQ values was evaluated, 14 mm implants exhibited significantly higher ISQ values than 8 mm implants across all combinations (*p* < 0.001). Cohen’s *d* values were high across all combinations (Table 3).

The effect of implant diameter was not consistent across all experimental conditions.

For Penguin RFA measurements, wider implants (4.8/5.0 mm) generally exhibited significantly higher ISQ values than narrow implants (3.5 mm) at a length of 14 mm. This difference was particularly pronounced in PCF30 for both T6 Standard (t = −9.648, *p* < 0.001, Cohen’s d = −5.570) and T6 Torq (t = −7.906, *p* < 0.001, Cohen’s d = −4.564). In contrast, diameter-related differences were not statistically significant in most 8 mm combinations (*p*> 0.05). A similar pattern was observed with Osstell ISQ, with wider implants generally showing higher ISQ values at 14 mm. The most pronounced difference was observed for T6 Standard implants in PCF30 (t = −11.180, *p* < 0.001, Cohen’s d = −6.455). Although significant diameter-related differences were also detected in some 8 mm combinations using Osstell ISQ, their effect sizes were relatively smaller.

Differences between the T6 and T6 Torq implant designs did not show a consistent pattern, with the results favoring either T6 or T6 Torq depending on the specific implant combination. Among the combinations showing statistically significant differences, effect sizes were generally large. The advantage of the T6 Torq design was most evident under conditions of low bone density and limited implant dimensions. Measurements obtained with both RFA devices showed that, in PCF15 blocks, T6 Torq implants with a diameter of 3.5 mm and a length of 8 mm exhibited significantly higher ISQ values than T6 implants (*p* < 0.001). Similarly, a significant difference in favor of the T6 Torq design was observed for 8 mm implants in the larger-diameter group in PCF15 blocks (*p* < 0.001).

Factorial ANOVA analyses performed for the Penguin RFA and Osstell ISQ datasets yielded highly consistent findings. For both devices, bone density exhibited the largest effect size, followed by implant length, implant diameter, and implant design. Detailed ANOVA results are presented in Table 4 and Table 5.

Regarding three-way interactions, significant effects were observed for Bone Density × Implant Design × Length (F = 62.296, *p* < 0.001, partial η^2^ = 0.438) and Bone Density × Diameter × Length (F = 10.667, *p* = 0.002, partial η^2^ = 0.118). For the Bone Density × Implant Design × Length interaction, the highest mean ISQ value was observed in the PCF30 group with the T6 Standard design and a length of 14 mm (77.7 ± 2.02), whereas the lowest value was recorded in the PCF15 group with the T6 Standard design and a length of 8 mm (57.7 ± 0.65). For the Bone Density × Diameter × Length interaction, the highest mean ISQ value was obtained in the PCF30 group with wider implants (4.8/5.0 mm) and a length of 14 mm (79.4 ± 0.79). In contrast, the Design × Diameter × Length (*p* = 0.786), Bone Density × Design × Diameter (*p* = 0.280), and four-way interactions (*p* = 0.417) were not statistically significant.

Regarding three-way interactions, significant effects were observed for Bone Density × Implant Design × Length (F = 36.271, *p* < 0.001, partial η^2^ = 0.312) and Bone Density × Diameter × Length (F = 20.156, *p* < 0.001, partial η^2^ = 0.201). For the Bone Density × Implant Design × Length interaction, the highest mean ISQ value was observed in the PCF30 group with the T6 Standard design and a length of 14 mm (76.5 ± 2.71), whereas the lowest value was recorded in the PCF15 group with the T6 Standard design and a length of 8 mm (56.5 ± 1.09). For the Bone Density × Diameter × Length interaction, the highest mean ISQ value was obtained in the PCF30 group with wider implants (4.8/5.0 mm) and a length of 14 mm (79.0 ± 0.85). In contrast, the Design × Diameter × Length (*p* = 0.327) and Bone Density × Design × Diameter (*p* = 0.232) interactions were not statistically significant.

Notably, the four-way interaction between Bone Density × Implant Design × Diameter × Length was statistically significant for the Osstell ISQ device (F = 5.288, *p* = 0.024, partial η^2^ = 0.062). Although the relatively small effect size suggests limited practical significance, this finding indicates that the combined effects of all four factors significantly influenced ISQ measurements. The results of the four-way interaction analysis for the Osstell ISQ device are presented in Table 6.

Among all combinations, the highest mean ISQ values were observed in the PCF30 group. The T6 Standard implant with a diameter of 4.8 mm and a length of 14 mm (79.0 ± 0.63) and the T6 Torq implant with a diameter of 5.0 mm and a length of 14 mm (79.0 ± 1.10) exhibited the highest values, with no statistically significant difference between them. In contrast, the lowest values were recorded in the PCF15 group, particularly for the T6 Standard implants with a diameter of 3.5 mm and a length of 8 mm (56.0 ± 1.10) and a diameter of 4.8 mm and a length of 8 mm (57.0 ± 0.89), both belonging to the lowest statistical group.

## 4. Discussion

Primary implant stability is a multifactorial phenomenon that reflects the mechanical interaction between the implant and the surrounding bone and is determined by the combined effects of bone quality and implant geometry [1,8,19]. ISQ values obtained by RFA provide a quantitative assessment of the stiffness of the implant-bone complex; however, these measurements may be influenced by several factors, including bone density, implant dimensions and macrodesign, bone-to-implant contact, transducer fixation, surrounding soft tissues, and surgical technique [20,21,22,23,24].

The present study evaluated the effects of bone density, implant design, implant diameter, and implant length on primary implant stability using two different RFA systems. The findings demonstrated that bone density was the dominant determinant of primary implant stability, followed by implant length, implant diameter, and implant design. In addition, both Osstell ISQ and Penguin RFA exhibited excellent measurement repeatability consistent with previous studies reporting high reproducibility of these RFA systems in clinical and experimental settings [7,25]. However, this finding should not be interpreted as evidence of agreement between devices, as the observed statistically significant inter-device differences suggest that the two systems may not be directly interchangeable.

Bone density exerted the greatest influence on primary stability in the present study. Regardless of implant design, diameter, or length, significantly higher ISQ values were obtained in PCF30 blocks than in PCF15 blocks. This finding supports the concept that primary stability is largely determined by the mechanical interaction between the implant and the surrounding bone. Haddad et al. [8] evaluated different implant site preparation techniques using PCF15 and PCF30 polyurethane blocks and reported higher primary stability in the higher-density material. Similarly, Raz et al. [26] observed higher stability values in dense bone models in their study evaluating different implant designs and lengths using Osstell and Penguin RFA devices. Diker et al. [7] also demonstrated a significant effect of bone density on ISQ values in implants with different macrodesigns and diameters placed in type 2 and type 3 artificial bone models. Clinical findings are broadly consistent with these experimental observations. Ostman et al. [27] reported higher ISQ values in the mandible than in the maxilla and demonstrated an association between bone quality and primary stability, whereas Bischof et al. [28] identified jaw and bone type as significant determinants of implant stability. These findings support the present observation that bone density had the largest effect size among the variables investigated. However, this observation should be interpreted cautiously, as each density condition was represented by a single polyurethane block without independent block-level replication.

Implant length was the second most influential factor affecting primary stability and exerted a more pronounced effect than implant diameter. Across the experimental combinations evaluated, 14 mm implants consistently demonstrated higher ISQ values than 8 mm implants. The increased bone-implant contact area associated with longer implants, together with their engagement within a greater volume of surrounding material, may contribute to this mechanical advantage. Farronato et al. [29] reported higher ISQ values with increasing implant length and substrate density, whereas Raz et al. [26] reported greater stability for longer implants, particularly under low-density bone conditions. Barikani et al. [30] also suggested that the contribution of implant length may become more pronounced in poor-quality bone. In contrast, Bischof et al. [28] found no significant influence of implant length on primary stability under clinical conditions. This discrepancy may partly reflect differences between homogeneous experimental models and natural bone. Heimes et al. [9] reported that although increasing implant length may enhance primary stability, this relationship does not appear to remain linear beyond approximately 12 mm.

Implant diameter also influenced ISQ values, with wider implants generally demonstrating greater primary stability than narrow implants. These findings are consistent with those of Diker et al. [7], Ostman et al. [27], and Tözüm et al. [31], who reported higher stability values for wider implants. However, Farronato et al. [29], found no significant influence of implant diameter on primary stability. In the present study, the effect of diameter appears to be less consistent than that of implant length, suggesting that its contribution may depend on interactions with other variables, particularly bone density, implant geometry, and implant length. Thus, although increasing implant diameter may contribute to primary stability, its mechanical benefit appears to be more context-dependent than that of implant length.

Although implant design showed the smallest overall effect size among the investigated variables, its influence became more evident under specific experimental conditions. In low-density bone and in groups with limited implant dimensions, the T6 Torq implant, characterized by a deeper and more aggressive thread geometry, demonstrated higher primary stability than the T6 Standard implant. This finding suggests that the influence of implant macrodesign is not independent but is instead closely related to the interaction between implant geometry and the surrounding bone. More aggressive thread configurations may enhance primary stability by increasing mechanical interlocking and improving engagement with low-density bone. Supachaiyakit et al. [17] found that implant design had a limited overall effect on stability but suggested that more aggressive thread configurations may offer an advantage under poor bone conditions. Ibrahim et al. [11] also demonstrated improved primary stability for implants with more aggressive thread characteristics. These findings support the concept that macrodesign may become particularly important when bone quality or implant dimensions are unfavorable. From a clinical perspective, the higher ISQ values observed for the T6 Torq implant in short and narrow configurations may be relevant to anatomically compromised situations where longer or wider implants cannot be placed. Under such circumstances, selecting an implant with a more aggressive macrodesign may provide an additional mechanical advantage for achieving adequate primary stability. However, this potential mechanical advantage was observed under controlled in vitro conditions and should not be interpreted as evidence of superior clinical performance, as long-term implant success depends on complex biomechanical and biological processes.

Antonelli et al. [32] investigated the effects of implant macrodesign and surgical site preparation on primary stability in low-density porcine bone using insertion torque and RFA measurements. Comparing conventional and magnetodynamic osteotomy techniques, they demonstrated that both implant design and surgical preparation influenced insertion torque and ISQ values, highlighting the importance of implant-bone mechanical interactions. These findings are consistent with the influence of implant macrodesign on primary stability observed in the present study while emphasizing the potential contribution of surgical preparation. Similarly, Gehrke et al. [33] reported significant differences in insertion torque and ISQ values between implant systems sharing a similar macrodesign, suggesting that additional implant-specific characteristics may influence primary stability. Notably, they found no significant correlation between insertion torque and ISQ, indicating that these parameters reflect distinct but complementary aspects of implant-bone mechanical behavior. In the present study, bone density was the predominant determinant of ISQ values, while implant dimensions and macrodesign also contributed to primary stability. However, the mechanical advantages observed under standardized experimental conditions may not directly translate into improved clinical outcomes. Furthermore, the absence of insertion torque measurements limits a more comprehensive biomechanical assessment of primary stability.

The use of homogeneous polyurethane blocks represents both a strength and a limitation of the present study. Their standardized density eliminates many of the anatomical and interindividual variations inherent to natural bone, allowing the controlled assessment of variables such as implant design, length, diameter, and substrate density. However, the low standard deviations observed under these controlled experimental conditions may have contributed to the exceptionally large Cohen’s d values. These effect sizes should therefore be interpreted cautiously and should not be assumed to reflect effects of comparable magnitude under clinical conditions, where greater biological and anatomical variability is expected. Furthermore, the use of a single polyurethane block for each density condition, without independent block-level replication, represents an additional methodological limitation. Additionally, these blocks do not fully reproduce the complex biological and mechanical characteristics of living bone. Kashtiban et al. [34] reported differences between implant stability measurements obtained in synthetic and natural bone, emphasizing that findings derived from polyurethane models should be interpreted cautiously when extrapolated to clinical conditions. Another limitation is that only two implant macrodesigns from a single manufacturer were evaluated, restricting the generalizability of the findings to other implant systems with different macrogeometries and surface characteristics. Furthermore, although both implant designs were evaluated using the same narrow diameter (3.5 mm), the wider implants differed slightly in diameter (4.8 mm and 5.0 mm). This 0.2 mm difference represents a potential confounding factor when comparing implant macrodesigns, as differences in primary stability cannot be attributed exclusively to thread geometry. Therefore, the independent effects of implant macrodesign should be interpreted with caution. In addition, implant stability was assessed immediately after placement under standardized in vitro conditions; therefore, the biological processes associated with osseointegration and changes in stability during healing could not be evaluated.

## 5. Conclusions

In conclusion, both Osstell ISQ and Penguin RFA demonstrated excellent measurement repeatability across the investigated experimental conditions. However, inter-device differences were more pronounced in narrow-diameter implants, suggesting that the two systems may not be directly interchangeable. Bone density was the principal determinant of primary implant stability, followed by implant length and implant diameter. Although implant design exerted the smallest overall effect, the more aggressive thread geometry of the T6 Torq implant provided a measurable stability advantage under low-density bone conditions when implant dimensions were limited. These findings indicate that implant selection should take into account not only implant dimensions but also local bone density and implant macrogeometry when attempting to optimize primary stability. The potential contribution of macrodesign may be particularly relevant in anatomically challenging situations in which the use of longer or wider implants is restricted. Future studies should incorporate bone models with distinct cortical and trabecular layers or natural bone specimens to better simulate clinical conditions. Comparisons involving implants with identical dimensions but different macrodesigns would help clarify the independent contribution of implant geometry to primary stability. Furthermore, evaluating a wider range of implant systems, bone densities, and surgical preparation protocols may improve the generalizability of the findings. Longitudinal clinical studies are also needed to investigate changes in implant stability throughout the healing period and to further evaluate the agreement between Osstell and Penguin RFA devices under clinical conditions.

## Figures and Tables

**Table 1:** ICC results.

Measurement Direction Buccal (B)/Lingual (L)	RFA Device	
	Penguin RFA	Osstell ISQ
B1	0.996 (0.994–0.997)	0.997 (0.996–0.998)
B2		
L1	0.997 (0.996–0.998)	0.997 (0.996–0.998)
L2		
B1-B2-L1-L2	0.999 (0.998–0.999)	0.999 (0.998–0.999)

**Table 2:** Differences between bone types across implant combinations.

RFA Device	Design	Diameter	Lenght	Bone Type		t	*p*	Cohen’s d
				PCF15	PCF30			
PenguinRFA	T6	3.5	8	57.5 ± 0.55	72.8 ± 0.75	−40.345	<0.001	−23.293
PenguinRFA	T6	3.5	14	65.7 ± 0.52	75.8 ± 0.41	−37.831	<0.001	−21.842
PenguinRFA	T6	4.8	8	57.8 ± 0.75	73.2 ± 0.98	−30.332	<0.001	−17.512
PenguinRFA	T6	4.8	14	66.8 ± 0.75	79.5 ± 0.84	−27.568	<0.001	−15.916
PenguinRFA	T6 Torq	3.5	8	60.7 ± 0.52	71.7 ± 0.82	−27.890	<0.001	−16.102
PenguinRFA	T6 Torq	3.5	14	64.8 ± 0.41	76.0 ± 0.63	−36.336	<0.001	−20.979
PenguinRFA	T6 Torq	5.0	8	61.2 ± 0.41	72.0 ± 1.26	−19.965	<0.001	−11.527
PenguinRFA	T6 Torq	5.0	14	66.8 ± 0.98	79.3 ± 0.82	−23.958	<0.001	−13.832
Osstell ISQ	T6	3.5	8	56.0 ± 1.10	70.5 ± 0.55	−29.000	<0.001	−16.743
Osstell ISQ	T6	3.5	14	63.3 ± 0.52	74.0 ± 0.89	−25.298	<0.001	−14.606
Osstell ISQ	T6	4.8	8	57.0 ± 0.89	72.3 ± 1.37	−23.000	<0.001	−13.279
Osstell ISQ	T6	4.8	14	65.8 ± 1.17	79.0 ± 0.63	−24.265	<0.001	−14.009
Osstell ISQ	T6 Torq	3.5	8	59.3 ± 0.82	70.7 ± 0.52	−28.735	<0.001	−16.590
Osstell ISQ	T6 Torq	3.5	14	63.5 ± 0.55	73.8 ± 0.75	−27.189	<0.001	−15.697
Osstell ISQ	T6 Torq	5.0	8	60.7 ± 1.21	70.2 ± 0.41	−18.208	<0.001	−10.512
Osstell ISQ	T6 Torq	5.0	14	65.3 ± 1.51	79.0 ± 1.10	−17.980	<0.001	−10.381

**Table 3:** Differences between implant lengths across implant combinations.

RFA Device	Bone Type	Design	Diameter	Lenght		t	*p*	Cohen’s d
				8	14			
PenguinRFA	PCF15	T6	3.5	57.5 ± 0.55	65.7 ± 0.52	−26.574	<0.001	−15.342
PenguinRFA	PCF15	T6	4.8	57.8 ± 0.75	66.8 ± 0.75	−20.708	<0.001	−11.956
PenguinRFA	PCF15	T6 Torq	3.5	60.7 ± 0.52	64.8 ± 0.41	−15.504	<0.001	−8.951
PenguinRFA	PCF15	T6 Torq	5.0	61.2 ± 0.41	66.8 ± 0.98	−13.038	<0.001	−7.528
PenguinRFA	PCF30	T6	3.5	72.8 ± 0.75	75.8 ± 0.41	−8.581	<0.001	−4.954
PenguinRFA	PCF30	T6	4.8	73.2 ± 0.98	79.5 ± 0.84	−12.017	<0.001	−6.938
PenguinRFA	PCF30	T6 Torq	3.5	71.7 ± 0.82	76.0 ± 0.63	−10.277	<0.001	−5.934
PenguinRFA	PCF30	T6 Torq	5.0	72.0 ± 1.26	79.3 ± 0.82	−11.931	<0.001	−6.888
Osstell ISQ	PCF15	T6	3.5	56.0 ± 1.10	63.3 ± 0.52	−14.832	<0.001	−8.563
Osstell ISQ	PCF15	T6	4.8	57.0 ± 0.89	65.8 ± 1.17	−14.700	<0.001	−8.487
Osstell ISQ	PCF15	T6 Torq	3.5	59.3 ± 0.82	63.5 ± 0.55	−10.381	<0.001	−5.993
Osstell ISQ	PCF15	T6 Torq	5.0	60.7 ± 1.21	65.3 ± 1.51	−5.916	<0.001	−3.416
Osstell ISQ	PCF30	T6	3.5	70.5 ± 0.55	74.0 ± 0.89	−8.174	<0.001	−4.719
Osstell ISQ	PCF30	T6	4.8	72.3 ± 1.37	79.0 ± 0.63	−10.847	<0.001	−6.262
Osstell ISQ	PCF30	T6 Torq	3.5	70.7 ± 0.52	73.8 ± 0.75	−8.497	<0.001	−4.906
Osstell ISQ	PCF30	T6 Torq	5.0	70.2 ± 0.41	79.0 ± 1.10	−18.508	<0.001	−10.686

**Table 4:** Factorial ANOVA results for the main effects and interactions assessed using the Penguin RFA device.

Source	Sum of Squares	df	Mean Square	F	*p*	Partial η^2^
Bone type	3675.375	1	3675.375	6534.000	<0.001	0.988
İmplant design	4.167	1	4.167	7.407	0.008	0.085
Implant diameter	51.042	1	51.042	90.741	<0.001	0.531
Implant length	864.000	1	864.000	1536.000	<0.001	0.950
Bone type × Length	13.500	1	13.500	24.000	<0.001	0.231
Design × Length	9.375	1	9.375	16.667	<0.001	0.172
Diameter × Length	28.167	1	28.167	50.074	<0.001	0.385
Bone type ×Design	24.000	1	24.000	42.667	<0.001	0.348
Bone type × Diameter	5.042	1	5.042	8.963	0.004	0.101
Design × Diameter	0.167	1	0.167	0.296	0.588	0.004
Bone type × Design × Length	35.042	1	35.042	62.296	<0.001	0.438
Bone type × Diameter × Length	6.000	1	6.000	10.667	0.002	0.118
Design ×Diameter × Length	0.042	1	0.042	0.074	0.786	0.001
Bone type × Design × Diameter	0.667	1	0.667	1.185	0.280	0.015
Bone type × Design × Diameter × Length	0.375	1	0.375	0.667	0.417	0.008
Error	45.000	80	0.562			
Total	4761.958	95				

**Table 5:** Factorial ANOVA results for the main effects and interactions assessed using the Osstell ISQ device.

Source	Sum of Squares	df	Mean Square	F	*p*	Partial η^2^
Bone type	3638.344	1	3638.344	4188.022	<0.001	0.981
Implant design	7.594	1	7.594	8.741	0.004	0.099
Implant diameter	123.760	1	123.760	142.458	<0.001	0.640
Implant length	834.260	1	834.260	960.300	<0.001	0.923
Bone type × Length	3.010	1	3.010	3.465	0.066	0.042
Design × Length	11.344	1	11.344	13.058	0.001	0.140
Diameter × Length	44.010	1	44.010	50.659	<0.001	0.388
Bone type × Design	29.260	1	29.260	33.681	<0.001	0.296
Bone type × Daimeter	8.760	1	8.760	10.084	0.002	0.112
Design * Diameter	2.344	1	2.344	2.698	0.104	0.033
Bone type × Design × Length	31.510	1	31.510	36.271	<0.001	0.312
Bone type × Diameter × Length	17.510	1	17.510	20.156	<0.001	0.201
Design × Diameter × Length	0.844	1	0.844	0.971	0.327	0.012
Bone type × Design × Diameter	1.260	1	1.260	1.451	0.232	0.018
Bone type × Design × Diameter × Length	4.594	1	4.594	5.288	0.024	0.062
Error	69.500	80	0.869			
Total	4827.906	95				

**Table 6:** Findings of the four-way interaction analysis using the Osstell ISQ device.

Bone Type	Design	Diameter	Length	Mean ± SS	F	*p*	Partial η^2^
PCF15	T6	3.5	8	56.0 ± 1.10 ^i^	5.288	0.024	0.062
			14	63.3 ± 0.52 ^f^			
		4.8	8	57.0 ± 0.89 ^i^			
			14	65.8 ± 1.17 ^e^			
	T6 Torq	3.5	8	59.3 ± 0.82 ^h^			
			14	63.5 ± 0.55 ^f^			
		5.0	8	60.7 ± 1.21 ^g^			
			14	65.3 ± 1.51 ^e^			
PCF30	T6	3.5	8	70.5 ± 0.55 ^d^			
			14	74.0 ± 0.89 ^b^			
		4.8	8	72.3 ± 1.37 ^c^			
			14	79.0 ± 0.63 ^a^			
	T6 Torq	3.5	8	70.7 ± 0.52 ^d^			
			14	73.8 ± 0.75 ^b^			
		5.0	8	70.2 ± 0.41 ^d^			
			14	79.0 ± 1.10 ^a^			

^a to i^: Values indicated by different letters are statistically significantly different from each other.

## Data Availability

The data presented in this study are available from the corresponding author upon reasonable request. The data are not publicly available because they contain detailed experimental datasets that were generated and analyzed during the current study and are available upon reasonable request from the corresponding author.

## Supplementary Materials

The following supporting information can be downloaded at: https://www.mdpi.com/article/10.3390/jcm15218626/s1, Table S1: ANOVA results and descriptive statistics for Penguin RFA device; Table S2: ANOVA results and descriptive statistics for Osstell device.
