# Supplementary material for: Evaluation of the Effects of Dental Implant Dimension and Design on Primary Implant Stability Using Different Stability Measurement Devices
Source: J Clin Med. 2026 Nov 5;15(21):8626. doi: 10.3390/jcm15218626 (PMC13763652; doi:10.3390/jcm15218626)
Supplement: Supplementary file 1 [file jcm-15-08626-s001.zip › jcm-4595976-supplementary.pdf]

Supplementary Table S1: ANOVA results and descriptive statistics for Penguin RFA device.

**Bone type**			**Mean ± SS**	**F**	***P***	**Partial η²**
PCF15			62.7 ± 3.70	6534.000	<0.001	0.988
PCF30			75.0 ± 3.07			
**Design**						
T6			68.6 ± 7.70	7.407	0.008	0.085
T6 Torq			69.1 ± 6.48			
**Diameter**						
3,5			68.1 ± 6.63	90.741	<0.001	0.531
5,0/4,8			69.6 ± 7.51			
**Lenght**						
8			65.9 ± 6.79	1536.000	<0.001	0.950
14			71.9 ± 6.07			
**Bone type**	**Design**	**Lenght**	**Mean ± SS**	**F**	***p***	**Partial η²**
PCF15	T6	8	57.7 ± 0.65[f]	62.296	<0.001	0.438
		14	66.3 ± 0.87[d]			
	T6 Torq	8	60.9 ± 0.51[e]			
		14	65.8 ± 1.27[d]			
PCF30	T6	8	73.0 ± 0.85[b]			
		14	77.7 ± 2.02[a]			
	T6 Torq	8	71.8 ± 1.03[c]			
		14	77.7 ± 1.87[a]			
**Bone type**	**Diameter**	**Lenght**	**Mean ± SS**	**F**	***p***	**Partial η²**
PCF15	3.5	8	59.1 ± 1.73[f]	10.667	0.002	0.118
		14	65.3 ± 0.62[e]			
	5.0/4.8	8	59.5 ± 1.83[f]			
		14	66,.8 ± 0.83[d]			
PCF30	3.5	8	72.3 ± 0.97[c]			
		14	75.9 ± 0.51[b]			
	5.0/4.8	8	72.6 ± 1.24[c]			
		14	79.4 ± 0.79[a]			
**Design**	**Diameter**	**Lenght**	**Mean ± SS**	**F**	***p***	**Partial η²**
T6	3.5	8	65.2 ± 8.03	0.074	0.786	0.001
		14	70.8 ± 5.33			
	4.8	8	65.5 ± 8.05			
		14	73.2 ± 6.66			
T6 Torq	3.5	8	66.2 ± 5.78			
		14	70.4 ± 5.85			
	5.0	8	66.6 ± 5.73			
		14	73.1 ± 6.58			
**Bone type**	**Design**	**Diameter**	**Mean ± SS**	**F**	***p***	**Partial η²**
PCF15	T6	3.5	61.6 ± 4.29	1.185	0.280	0.015
		4.8	62.3 ± 4.75			
	T6 Torq	3.5	62.8 ± 2.22			
		5.0	64.0 ± 3.05			
PCF30	T6	3.5	74.3 ± 1.67			
		4.8	76.3 ± 3.42			
	T6 Torq	3.5	73.8 ± 2.37			
		5.0	75.7 ± 3.96			

[a,b,c]: Values indicated by different letters are statistically significantly different.

Supplementary Table S2: ANOVA results and descriptive statistics for Osstell device

Bone type			Mean ± SS	F	*P*	Partial η²
PCF15			61.4 ± 3.64	4188.022	<0.001	0.981
PCF30			73.7 ± 3.48			
**Design**						
T6			67.3 ± 7.79	8.741	0.004	0.099
T6 Torq			67.8 ± 6.48			
**Diameter**						
3,5			66.4 ± 6.47	142.458	<0.001	0.640
5,0/4,8			68.7 ± 7.63			
**Lenght**						
8			64.6 ± 6.62	960.300	<0.001	0.923
14			70.5 ± 6.42			
**Bone type**	**Design**	**Lenght**	**Mean ± SS**	**F**	***p***	**Partial η²**
PCF15	T6	8	56.5 ± 1.09[e]	36.271	<0.001	0.312
		14	64,.6 ± 1.56[c]			
	T6 Torq	8	60.0 ± 1.21[d]			
		14	64.4 ± 1.44[c]			
PCF30	T6	8	71.4 ± 1.38[b]			
		14	76.5 ± 2.71[a]			
	T6 Torq	8	70.4 ± 0.51[b]			
		14	76.4 ± 2.84[a]			
**Bone type**	**Diameter**	**Lenght**	**Mean ± SS**	**F**	***p***	**Partial η²**
PCF15	3.5	8	57.7 ± 1.97[g]	20.156	<0.001	0.201
		14	63.4 ± 0.51[e]			
	5.0/4.8	8	58.8 ± 2.17[f]			
		14	65.6 ± 1.31[d]			
PCF30	3.5	8	70.6 ± 0.51[c]			
		14	73.9 ± 0.79[b]			
	5.0/4.8	8	71.3 ± 1.48[c]			
		14	79.0 ± 0.85[a]			
**Design**	**Diameter**	**Lenght**	**Mean ± SS**	**F**	***p***	**Partial η²**
T6	3.5	8	63.3 ± 7.62	0.971	0.327	0.012
		14	68.7 ± 5.61			
	4.8	8	64.7 ± 8.08			
		14	72.4 ± 6.93			
T6 Torq	3.5	8	65.0 ± 5.95			
		14	68.7 ± 5.43			
	5.0	8	65.4 ± 5.04			
		14	72.2 ± 7.25			
**Bone type**	**Design**	**Diameter**	**Mean ± SS**	**F**	***p***	**Partial η²**
PCF15	T6	3.5	59.7 ± 3.92	1.451	0.232	0.018
		4.8	61.4 ± 4.72			
	T6 Torq	3.5	61.4 ± 2.27			
		5.0	63.0 ± 2.76			
PCF30	T6	3.5	72.3 ± 1.96			
		4.8	75.7 ± 3.63			
	T6 Torq	3.5	72.3 ± 1.76			
		5.0	74.6 ± 4.68			

[a,b,c]: Values indicated by different letters are statistically significantly different.
